# A Multi-Marker Test for Analyzing Paired Genetic Data in Transplantation

**DOI:** 10.3389/fgene.2021.745773

**Published:** 2021-10-13

**Authors:** Victoria L. Arthur, Zhengbang Li, Rui Cao, William S. Oetting, Ajay K. Israni, Pamala A. Jacobson, Marylyn D. Ritchie, Weihua Guan, Jinbo Chen

**Affiliations:** ^1^ Department of Biostatistics, Epidemiology, and Informatics, University of Pennsylvania Perelman School of Medicine, Philadelphia, PA, United States; ^2^ Departments of Statistics, Central China Normal University, Wuhan, China; ^3^ Division of Biostatistics, School of Public Health, University of Minnesota, Minneapolis, MN, United States; ^4^ Department of Experimental and Clinical Pharmacology, College of Pharmacy, University of Minnesota, Minneapolis, MN, United States; ^5^ Minneapolis Medical Research Foundation, Minneapolis, MN, United States; ^6^ Department of Medicine, Hennepin County Medical Center, Minneapolis, MN, United States; ^7^ Department of Epidemiology and Community Health, University of Minnesota, Minneapolis, MN, United States; ^8^ Department of Genetics, Perelman School of Medicine, University of Pennsylvania, Philadelphia, PA, United States

**Keywords:** transplant genetics, multi-marker testing, joint testing, genetic matching scores, paired genetic data

## Abstract

Emerging evidence suggests that donor/recipient matching in non-HLA (human leukocyte antigen) regions of the genome may impact transplant outcomes and recognizing these matching effects may increase the power of transplant genetics studies. Most available matching scores account for either single-nucleotide polymorphism (SNP) matching only or sum these SNP matching scores across multiple gene-coding regions, which makes it challenging to interpret the association findings. We propose a multi-marker Joint Score Test (JST) to jointly test for association between recipient genotype SNP effects and a gene-based matching score with transplant outcomes. This method utilizes Eigen decomposition as a dimension reduction technique to potentially increase statistical power by decreasing the degrees of freedom for the test. In addition, JST allows for the matching effect and the recipient genotype effect to follow different biological mechanisms, which is not the case for other multi-marker methods. Extensive simulation studies show that JST is competitive when compared with existing methods, such as the sequence kernel association test (SKAT), especially under scenarios where associated SNPs are in low linkage disequilibrium with non-associated SNPs or in gene regions containing a large number of SNPs. Applying the method to paired donor/recipient genetic data from kidney transplant studies yields various gene regions that are potentially associated with incidence of acute rejection after transplant.

## 1 Introduction

Transplant matching usually focuses on non-genetic factors related to the donor, the recipient, or the graft itself, such as recipient age, donor sex, or organ size. Genetic matching in transplant has been limited to the human leukocyte antigen (HLA) region of the genome in the past, as this region codes for immune related genes that may lead to the recipient recognizing the allograft as non-self and mounting an immune response against it (Reddy et al., 2013; Hernandez-Fuentes et al., 2018). Although HLA matching reduces the risk of allograft rejection, it is not enough to prevent allograft rejection, even in the case of transplant between HLA-identical siblings (Grafft et al., 2010; Zanoni and Kiryluk, 2020). More recent transplant genetic studies have identified gene regions outside of the HLA region that may act as genetic modifiers for transplant outcomes (Almoguera et al., 2014; Yang and Sarwal, 2017; Steers et al., 2019; Farouk et al., 2020; Marin et al., 2020; Reindl-Schwaighofer et al., 2020). These so-called minor histocompatibility antigens may be important regions of interest to examine further in order to improve transplant outcomes.

Several studies have found evidence suggesting that donor and recipient genetic mismatch in these non-HLA regions could impact transplant outcomes. Zhang et al. (2020) showed that non-HLA donor/recipient (D/R) genetic differences were significantly associated with long-term graft survival in kidney transplant. Pineda et al. (2017) found a significantly increased number of D/R mismatched variants in the group of kidney transplant recipients with antibody-mediated rejection (AMR) compared to the group with no rejection, and they were able to identify 16 gene regions with multiple SNPs associated with AMR. Steers et al. (2019) utilized a genomic-collision model, in which a recipient who is homozygous for a deletion tagging allele obtains a transplant from a non-homozygous donor and were able to find a single polymorphism located in the LIMS1 locus with an increased hazard for rejection for D/R pairs with the collision genotype. In addition, previous work on single nucleotide polymorphism (SNP) matching in transplant found that utilizing D/R matching scores in association analyses led to discovery of SNPs potentially associated with acute rejection after liver transplant. Joint testing of these scores with the recipient genotype also suggested that the scores were measuring some aspect other than the combination of the recipient and donor genotype, since there were cases where the score was associated with transplant outcome, but the recipient and donor genotypes were not (Arthur et al., 2020). In order to improve power, increase interpretability and reproducibility of association signals, and facilitate follow-up functional studies, it is of interest to extend these single SNP methods to a multi-marker framework.

Many multi-marker methods have been proposed in the literature for assessing the association of multiple genetic markers within a gene region. Depending on how association information for individual markers is aggregated, they can largely be classified into three groups. In the first group, each test is based on combining *p*-values from tests of individual markers (Li et al., 2011; Chen et al., 2012; Mishra and Macgregor, 2015). Members in the second group can be seen as some quadratic form that combines statistics for testing marginal associations with each marker (Pan 2011). Well-known examples include Hotelling’s T^2^ statistic (Fan & Knapp, 2003), genomic-distance based regression (Wessel and Schork, 2006), variance component (VC) or kernel machine regression based tests (Tzeng and Zhang 2007; Pan 2009; Wu et al., 2010; Wu et al., 2011), the use of weighted genetic risk scores (Li et al., 2009; Iribarren et al., 2018), and the C-alpha test (Neale et al., 2011). These tests have also been extended using the framework of functional or mixed effects models (Fan et al., 2013; Chen et al., 2019; Chiu et al., 2019). In the third group, each test assesses associations between the phenotype variable and some form of aggregated genotype data. For example, the principal component regression (PCR) method (Gauderman et al., 2007; Wang and Abbot 2008; Chen and Qin, 2010; de Leeuw et al., 2015) tests the significance of top principal components of centered multi-marker genotype data, and Wang and Elston (2007) test similarly collapsed variables obtained via Fourier transformations. Similarly to tests in the second group, tests in this group have been built upon using functional data analysis methods (Luo et al., 2011; Luo et al., 2013). PCR and VC methods have been shown to generally have competitive statistical power (Ballard et al., 2010; Pan 2011; Liu et al., 2020). The connections among some of these methods have been studied in the literature (Schaid 2010; Bacanu 2012).

Only a few multi-marker D/R matching scores have been utilized for association analysis of transplant genetics data. The allogenomics mismatch score (AMS) is based on the hypothesis that observing the coding regions of both the recipient and donor genomes can help identify the number of potentially incompatible amino acids between the pair (Mesnard et al., 2016). The AMS is defined as the sum of amino acid mismatch contributions across all SNPs in the exome. A negative linear association was observed between the AMS and estimated glomerular filtration rate at 36 months post-transplant, suggesting that the AMS may be correlated with long term kidney graft function (Mesnard et al., 2016). A second method defined variant mismatch as any allele difference between the paired recipient and donor genomes (Pineda et al., 2017). This study found that the total number of D/R variant mismatches prior to transplant was significantly higher in the recipient group that developed antibody-mediated rejection versus the group with no rejection. The final method defined SNP mismatch as the donor carrying an allele not present in the recipient genome. These individual mismatches were then summed over all non-synonymous SNPs (nsSNPs) in the genome. After fitting a multivariate model that adjusted for HLA eplet mismatch, the degree of nsSNP mismatch was independently associated with graft loss (Reindl-Schwaighofer et al., 2019). While these methods were able to find some association between genome-wide mismatch and transplant outcomes, the results are difficult to interpret due to the scores spanning the entire genome. By extending existing gene-based test ideas to use both the recipient and donor genotype information, we have the potential to increase the power and interpretability over single SNP and whole genome-wide scoring methods.

Here we propose a new method called the “Joint Score Test (JST).” JST is built upon marginal likelihood scores for testing multiple SNP effects and a gene-based D/R matching score. JST uses only the most informative linear combinations of single recipient SNP marginal likelihood scores to allow for an increase in statistical power. JST also allows for flexible adjustment for covariates and therefore it can maintain nominal type-I error rates in the presence of population stratification.

We organize our paper as follows. First, we discuss the underlying model and the construction of JST. Then we present the results of extensive simulation studies. Third, we utilize JST in an association analysis of kidney transplant data. Finally, we discuss the benefits and potential limitations of the method.

## 2 Materials and Methods

JST jointly tests whether recipient genotype SNPs or a gene-based D/R matching score are associated with the transplant phenotype of interest.

### 2.1 Notation

Suppose that genotype data for *m* SNPs in a genomic region of interest are available for *n* transplant D/R pairs. Let $\;X_{ij}^{R}$ and $X_{ij}^{D}$ be the numerical coding of the genotype of the *j*th marker for the *i*th recipient or donor, respectively (i=1,…,n; j=1,…,m) , which can be the number of minor alleles or another numerical coding, and let $W_{ik}$ be the *k*th covariate of D/R pair *i*. We consider a regression setting with continuous or categorical outcomes, *Y*_*i*_, and our generalized linear model (GLM) is of the form

$$g\left(\mu_{i}\right)=\;\alpha_{0}\;+\;W_{i}\alpha +\;X_{i}\beta +\;Z_{i}\gamma ,\;$$

where *g* () is the link function, $\mu_{i}=E\left(Y_{i}|W_{i},\;X_{i},\;\;Z_{i}\right)$ , $W_{i}=\left(W_{i1},...,W_{iK}\right)$ is the vector of *K* covariates for D/R pair *i* with regression coefficients $\alpha ={\left(\alpha_{1},\ldots ,\alpha_{k}\right)}^{T}.\;$ Let $\;X_{i}=\left(X_{i1}^{R},X_{i2}^{R},...,X_{im}^{R}\right)$ denote the genotype vector of *m* SNPs for recipient *i* with regression coefficients $\beta ={\left(\beta_{1},\ldots ,\beta_{m}\right)}^{T},$ and let $Z_{i}$ denote the single gene-based genetic matching score value for D/R pair *i* with regression coefficient γ . Note that W  may include principal components (PCs) for describing population substrata. We are interested in jointly testing the null hypotheses

$$H_{0}:\;\beta =0\;\text{and}\;\gamma =0$$

### 2.2 Gene-Based Scores

In general, each gene-based score can be written in the form

$$Z_{i}=\overset{m}{\underset{j=1}{\sum }}D\left(X_{ij}^{D}\;,\;X_{ij}^{R}\;\right)$$

where D $\left(X_{ij}^{D}\;,\;X_{ij}^{R}\;\right)$ represents a function defining a distance between the donor and recipient genotypes. We emphasize that the donor and recipient SNPs used in the distance calculations are not necessarily the same as those present in the recipient genotype main effects vector $\;X_{i}$ , and thus can be greater or fewer in number. We will focus on four different single SNP distance functions.

### 2.3 Single SNP Distance Scores

The first score, the identity-by-state (IBS) mismatch distance function, is defined as

$$D_{IBS}=|X_{ij}^{D}-\;X_{ij}^{R}\;|\;$$

assuming diallelic SNPs. This function is based on the degree of identity-by-state between the donor and recipient genotypes, measuring the number of alleles the pair shares at a SNP. IBS has previously been used as a kernel function in the sequence kernel association test (Wu et al., 2011) and in a kernel machine approach to test multiple genetic markers in association with quantitative traits (Kwee et al., 2008).

The second score considered, the incompatibility distance function, is calculated as

$$D_{Incomp}=\;\{\begin{array}{c} 1\;if\;X_{ij}^{D}\neq X_{ij}^{R}\;\;\\ 0\;otherwise. \end{array}$$

This distance metric has been utilized in a kidney transplant study, where single SNP incompatibility and a genome-wide sum of this measure were found to be associated with antibody-mediated rejection (Pineda et al., 2017). In addition, a similar score was utilized in mother/child pairs in a genetic study of pre-eclampsia (PE), where they found SNPs from three candidate gene regions to be nominally associated with PE (Parimi et al., 2008).

The third score, the Allogenomics Mismatch Score (AMS) distance function, is defined as

$$D_{AMS}=\;\sum_{a\;\in \;X_{ij}^{D}}\{\begin{array}{c} 0\;if\;a\;\in \;X_{ij}^{R}\\ 1\;otherwise, \end{array}$$

where *a* denotes alleles of a genotype (Mesnard et al., 2016). The underlying hypothesis of this method states that examining the difference between transplant donor and recipient alleles in coding regions of the genome can give insight into which amino acids coded by the donor would present as non-self to the recipient immune system, potentially leading to allograft damage.

The fourth score, the binary mismatch score, is based off a simplification of the AMS which assigned a score of 1 for all SNPs where the donor genotype contained an allele that was not present in the recipient genotype and a score of 0 otherwise (Reindl-Schwaighofer et al., 2019). The single SNP distance function can be defined as

$$D_{MM}=\;\{\begin{array}{c} 1\;if\;\exists \;a\in G_{dl}\;such\;that\;a\notin G_{rl}\\ 0\;otherwise \end{array}$$

### 2.4 A New Multi-Marker Test Statistic for Paired Transplant Data

In this section we will focus on deriving our JST test statistic for a binary outcome, Y. Additional derivation for a binary and a continuous outcome is provided in the Online Resource. Let ${\hat{p}}_{1}\left(W_{i}\right)\equiv p\left(Y_{i}=1|W_{i};{\hat{\alpha }}_{0};\hat{\alpha }\right)$ denote the predicted probability of $Y_{i}=1$ based on the null model

$$logit\;Pr\left(Y_{i}=1\text{|}W_{i};\alpha_{0},\;\alpha \right)=\alpha_{0}+\overset{K}{\underset{k=1}{\sum }}W_{ik}\alpha_{k}\equiv \alpha_{0}+W_{i}\alpha .$$

Here ${\hat{\alpha }}_{0}$ and $\hat{\alpha }$ are the maximum likelihood estimates of $\alpha_{0}$ and α . Additionally, we let ${\hat{X}}_{ij}^{R}$ denote the fitted value for the *j*th SNP genotype for recipient *i* from a weighted linear regression model $X_{ij}^{R}=\;\theta_{0}+\;\overset{K}{\underset{k=1}{\sum }}W_{ik}\theta_{k}+\;\varepsilon_{X}$ and let ${\hat{Z}}_{i}$ denote the fitted value for the gene-based genetic matching score of D/R pair *i* from a weighted linear regression model $Z_{i}=\;\tau_{0}+\;$ $\overset{K}{\underset{k=1}{\sum }}W_{ik}\tau_{k}+\;\varepsilon_{Z}$ . In both cases, the weights are ${\hat{p}}_{1}(W_{i})\left\{1-\;{\hat{p}}_{1}\left(W_{i}\right)\right\}$ for recipient *i* or D/R pair *i*. As derived in the Online Resource, the likelihood score for testing the marginal association with the *j*th recipient SNP is equivalent to

$$U_{j}^{R}=\;\overset{n}{\underset{i=1}{\sum }}\left(X_{ij}^{R}-{\hat{X}}_{ij}^{R}\right)\left\{Y_{i}-\;\;{\hat{p}}_{1}\left(W_{i}\right)\right\}\equiv \;\overset{n}{\underset{i=1}{\sum }}Q_{ij}^{R}$$

Similarly, the likelihood score for testing the marginal association with the gene-based D/R SNP genetic matching score is equivalent to

$$U^{S}=\;\overset{n}{\underset{i=1}{\sum }}\left(Z_{i}-{\hat{Z}}_{i}\right)\left\{Y_{i}-\;\;{\hat{p}}_{1}\left(W_{i}\right)\right\}\equiv \;\overset{n}{\underset{i=1}{\sum }}Q_{i}^{S}$$

Denote the vector of scores for all *m* recipient genotype SNPs and the gene-based genetic matching score as U , $U={\left(U_{1}^{R},...,U_{m}^{R},\;U^{S}\right)}^{T}$ , let $B_{i}=\left(X_{i},Z_{i}\right)$ and ${\hat{B}}_{i}=\left({\hat{X}}_{i},{\hat{Z}}_{i}\right)$ . Then ***U*** can be written into the matrix form,

$$U={\left(B-\hat{B}\right)}^{T}\left\{Y-{\hat{p}}_{1}\right\}$$

which is asymptotically distributed as a (m+1)  dimensional normal random variable with variance-covariance matrix $V=nQ^{T}Q$ where $Q=\left(Q^{R},Q^{S}\right)$ . The element of ***V*** at position (a,b),  a=1,...,(m+1) and b=1,...,(m+1), is estimated as $n\overset{n}{\underset{i=1}{\sum }}Q_{ia}^{T}Q_{ib}.$ A Hotelling’s *T* ^2^ statistic can be constructed as $nU^{T}{\hat{V}}^{-1}U,$ which asymptotically follows a Chi-squared distribution with (m+1) degrees of freedom. It is well known that the Hotelling’s *T* ^2^ statistic has low power when (m+1) is large, and that eliminating ${\hat{V}}^{-1}$ from the test statistic could lead to an improvement in power (Bai and Saranadasa, 1996). Along this line, a squared score test (Pan 2009, referred to as “SSU”) and a kernel-machine based test with the linear kernel (Wu et al., 2010, referred to as “SKAT”) have increased power for testing multiple marker main effects. These methods do not distinguish between the matching score effects and recipient SNP effects, however, which may have different underlying biological mechanisms. The sequence kernel association test (SKAT) method, for example, assumes that β and γ have the same underlying variance component (Wu et al., 2011), which therefore does not distinguish recipients’ SNP main effects and the effect of the matching score.

Here we propose a new statistic as follows. V  can be decomposed as

$$\left[\begin{array}{cc} \;V^{R} & C^{\mathrm{RS}}\\ C^{\mathrm{SR}} & V^{S} \end{array}\right]=\left[\begin{array}{cc} Var\left(U^{R}\right) & Cov\left(U^{R},\;U^{S}\right)\\ Cov\left(U^{S},\;U^{R}\right) & Var\left(U^{S}\right) \end{array}\right]$$

Our statistic is based on Eigen decomposition of the sample variance-covariance matrix ${\hat{V}}^{R}.\;\;$ Let $A=\left[a_{1},a_{2},\ldots ,a_{m}\right]$ denote a m × m matrix with the *p*th column being the *p*th eigenvector of ${\hat{V}}^{R},\;$ and $\left(\lambda_{1},\lambda_{2},\ldots ,\lambda_{m}\right),\;\lambda_{1}\geq \ldots \geq \lambda_{m},$ denote the corresponding eigenvalues. We extract the first *s* (*s* < m ) principal components (PCs, the choice of *s* is discussed below). Let $A_{s}=\left[a_{1},a_{2},\ldots ,a_{s}\right].$ Define $U^{\mathrm{PR}}$ as the vector of ${\left(U^{R}\right)}^{T}a_{l}/\sqrt{\;\lambda_{l}}$ , l=1,2,…,s. Our test statistic is constructed based on $U^{P}=\left(U^{\mathrm{PR}},U^{S}\right)$ .

$${\left(\begin{array}{c} U^{\mathrm{PR}}\\ U^{S} \end{array}\right)}^{T}{\left[\begin{array}{cc} I_{s\times s} & Côv\left(U^{\mathrm{PR}},\;U^{S}\right)\\ Côv\left(U^{S},\;U^{\mathrm{PR}}\right) & Vâr\left(U^{S}\right) \end{array}\right]}^{-1}\left(\begin{array}{c} U^{\mathrm{PR}}\\ U^{S} \end{array}\right)$$

where $I_{s\times s}$ is the *s* by *s* identity matrix.

We can show that this statistic is asymptotically distributed as a Chi-squared random variable with *s+1* degrees of freedom under the null. Therefore, $I_{s\times s}$ is there under the null due to the orthogonality between the eigenvectors. Under the alternative hypothesis, this statistic is distributed as a non-central Chi-squared random variable with *s+1* degrees of freedom and non-centrality parameter that is equal to its value.

### 2.5 Selection of s for JST

Choosing the number of PCs to retain (*s*) is always a difficult task in principal component analysis. One common method is to choose the number of PCs to keep based on a predefined percentage of total variance explained. We utilize this method in our simulation studies, looking at a range of 65–99% total variance explained by the retained PCs.

### 2.6 Simulations

Simulation studies were conducted to assess type I error and power levels of the JST, as well as to determine the number of principal components to maintain after Eigen decomposition, *s*.

#### 2.6.1 Simulation Study Design

Datasets were sampled from 1,000 Genomes Phase 3 reference using HapGen2 (Su, Marchini and Donnelly, 2011). Briefly, subsets of the reference data were created based on three gene regions starting and ending positions, *NAT2*, *CHI3L2*, and *ASAH1*. These genes were chosen due to their differing number of SNPs and LD structures (Supplementary Figure S1). SNPs with minor allele frequency less than 0.05 were excluded from analyses. The subset reference data was then sampled with HapGen2 to generate 2*n* control individuals which were then paired into *n* donor/recipient pairs. A small sample size, *n = 500*, and a large sample size, *n = 1,000*, were considered. Recipient and donor genotype information was then extracted from these sampled datasets and used to calculate gene-based scores. A total of 5,000 simulations were conducted for each gene region and sample size combination.

For type I error analysis, null phenotypes were generated using the model

$$logit\;Pr\left(Y_{i}=1|W\right)=\alpha_{0}+0.5W_{1}+0.5W_{2}$$

for binary outcome $Y_{i}$ , and using the model

$$Y_{i}=0.5W_{1}+0.5W_{2}+\;\varepsilon$$

for continuous outcome $Y_{i}$ , where $W_{1}$ is a binary covariate taking a value of either 0 or 1 with probability 0.5, $W_{2}$ is a continuous covariate drawn from a standard Normal distribution, and ε is an error term drawn from a standard Normal distribution.

For power analyses, phenotypes were generated using the model

$$logit\;Pr\left(Y_{i}=1|W,X,Z\right)=\;\alpha_{0}\;+\;0.5W_{1}+0.5W_{2}+\;X_{i}\beta +\;Z_{i}\gamma ,\;$$

for binary outcome $Y_{i}$ , or using the model

$$Y_{i}=\;0.5W_{1}+0.5W_{2}+\;X_{i}\beta +\;Z_{i}\gamma \;+\;\varepsilon$$

for continuous outcome $Y_{i}$ . For both models, a variety of true associations were tested, where either recipient genotype SNPs were associated ( β ≠0, γ=0 ), or D/R matching was associated ( β=0, γ≠0) . When recipient genotype SNPs were associated, we considered scenarios in which 5, 15, or 25% of the SNPs in the gene region were truly associated with outcome, and this group of SNPs was either in high linkage disequilibrium (LD) or low LD. When D/R matching was associated, we considered scenarios in which 5, 15, 25, 50, 75, or 100% of the SNPs in the gene region were important to match between D/R. For cases where less than 100% of the SNPs were important to match, we included only the associated SNPs in the summed matching score when deriving phenotypes, and then utilized the full gene-based matching score for testing. Similarly to the recipient genotype SNPs, these groups of matched SNPs were either in high or low LD with one another. We considered a small, 0.14, medium, 0.41, and a large, 0.69, effect size resulting in odds ratios of 1.25, 1.50, and 2.00. Prevalence of the binary outcome $Y_{i}$ ranged from 5 to 20% in order to see the effects of rare versus common outcomes. In addition, several values of *s* were examined, accounting for 65–99% of total variance explained by the principal components, to determine its effect on type I error and power levels. All analyses were run using R (v4.0, R Core Team, 2021). Code to run all simulations can be found online (https://github.com/arthurvickie/Multi-Marker_Method).

#### 2.6.2 Comparison to Existing Methods

In addition to testing the type I error and power levels of JST, we compared our method with a standard GLM and the SKAT. For all comparisons, phenotype generation was the same as for JST. We used the same *n* x (*m+1*) matrix of combined recipient genotype SNPs and gene-based D/R matching score as input as was used for JST. For our standard GLM, we fit separate models under the null and alternative hypotheses respectively, and then calculated the likelihood ratio test (LRT) statistic using the lrtest function from the lmtest (v0.9-37, Zeileis and Hothorn, 2002) package in R or the score test statistic using the anova function in R. SKAT analysis was performed using the unweighted linear kernel and the unweighted IBS kernel as implemented in the SKAT (v1.3.2.1, Lee et al., 2017) package in R.

### 2.7 Real Data Analysis

#### 2.7.1 Sample Information

Kidney transplant data was collected from two cohorts: Deterioration of Kidney Allograft Function (DeKAF, 2005–2011, NCT00270712) Genomics Study and Genomics of Kidney Transplantation (GEN-03, 2012–2016, NCT01714440) study. Genotypes from the DeKAF cohort (*n* = 784 donor-recipient pairs) were determined with the AFR-AMR Axiom chip (Affymetrix, Santa Clara, CA) (Hoffmann et al., 2011), which contains 837,930 variants. Genotyping of GEN-03 cohort (*n* = 404 donor-recipient pairs) was performed on a custom exome-plus Affymetrix TxArray SNP chip (Li et al., 2015), which contains approximately 782,000 variants. Genotype calling was performed in one batch on the Affymetrix Genotyping Console v4.0 using the GT1 algorithm, which is based on BRLMM-P (Affymetrix, Santa Clara, CA). Genotyping details can be found in our previous paper (Oetting et al., 2016). Non-Caucasian recipients were excluded from this study. In both data sets the outcome of interest incidence of acute rejection (AR) after transplant (161 cases in DeKAF cohort and 50 cases in GEN-03 cohort), was coded as a binary variable. AR was defined as time to first T-cell, antibody mediated, or mixed T-cell and antibody mediated rejection post-transplant as determined by the enrolling center and treating physician. Rejection was biopsy confirmed in ∼96% of the cases.

#### 2.7.2 Statistical Analysis

The common SNPs in the two cohorts were grouped by physical locations within 23,062 genome-wide genes (GRCh38. p13) and then analyzed using both JST with *s* = 85% variance explained and SKAT with an unweighted IBS kernel. Covariates were included for recipients’ age, gender, PRA status, prior non-kidney transplantation, and an indicator for the cohort membership. *p*-values were adjusted for multiple comparisons using the Benjamini-Hochberg procedure to control false discovery rate (FDR).

## 3 Results

### 3.1 Results of Simulation Studies

A subset of the total simulation results is presented for both type I error testing and power testing. In all scenarios, results were similar for all combinations of gene, sample size, and any additional variables tested. Table 1 is based on scenarios using *NAT2* with 500 D/R pairs, and all figures are based on 1000 D/R pairs, using data from either *NAT2* or *CHI3L2*.

**TABLE 1:** Results of Type I Error simulations for the gene *NAT2* with 500 D/R pairs. Method refers to one of the four multi-marker methods used for testing. Score refers to the gene-based score that was fit as part of the modeling. The columns Prevalence 20, 10, and 5% give results for binary outcome *Y,* and the continuous column gives results for continuous outcome *Y*. JST stands for Joint Score Test, with *s* value (percent of variance explained by the PCs) indicated in parentheses. Results for *s* value between 70 and 95% were similar to those for *s* = 65%. SKAT stands for Sequence Kernel Association Test. SKAT (Linear) refers to using SKAT with an unweighted linear kernel. SKAT (IBS) refers to fitting SKAT with an unweighted IBS kernel. GLM stands for Generalized Linear Model.

Method	Score	Prevalence			Continuous
		20%	10%	5%	
JST (s = 65%)	IBS	0.05	0.05	0.06	0.05
	Incompatibility	0.05	0.04	0.05	0.05
	AMS	0.05	0.05	0.06	0.04
	Binary MM	0.05	0.05	0.06	0.04
JST (s = 99%)	IBS	0.04	0.05	0.01	0.04
	Incompatibility	0.05	0.05	0.01	0.04
	AMS	0.04	0.05	0.01	0.04
	Binary MM	0.04	0.05	0.01	0.03
SKAT Linear	IBS	0.05	0.05	0.05	0.05
	Incompatibility	0.05	0.05	0.05	0.05
	AMS	0.05	0.05	0.05	0.05
	Binary MM	0.05	0.05	0.05	0.05
SKAT IBS	IBS	0.05	0.05	0.04	0.05
	Incompatibility	0.06	0.05	0.05	0.05
	AMS	0.05	0.05	0.05	0.05
	Binary MM	0.05	0.05	0.05	0.05
GLM	IBS	0.05	0.07	0.10	0.06
	Incompatibility	0.06	0.07	0.11	0.06
	AMS	0.05	0.08	0.10	0.05
	Binary MM	0.05	0.07	0.10	0.06

#### 3.1.1 Type I Error

Table 1 shows the results of type I error rates for joint testing. For the proposed JST method, the type I error rate was around 0.05 for all combinations of outcome prevalence and fitted gene-based score. Type I error rates are nominal for *s* values between 65 and 90%, with some conservative error rates seen with *s* = 99% for lower outcome prevalence. Similar results can be seen for the SKAT method using either the linear or IBS kernel. The standard score test based on generalized linear model tends to have inflated type I error values when the outcome is binary, ranging from around 5–11%. When the outcome is continuous, the type I error is slightly inflated, at 6%.

#### 3.1.2 Power—Recipient Genotype SNPs Associated

**FIGURE 1:**
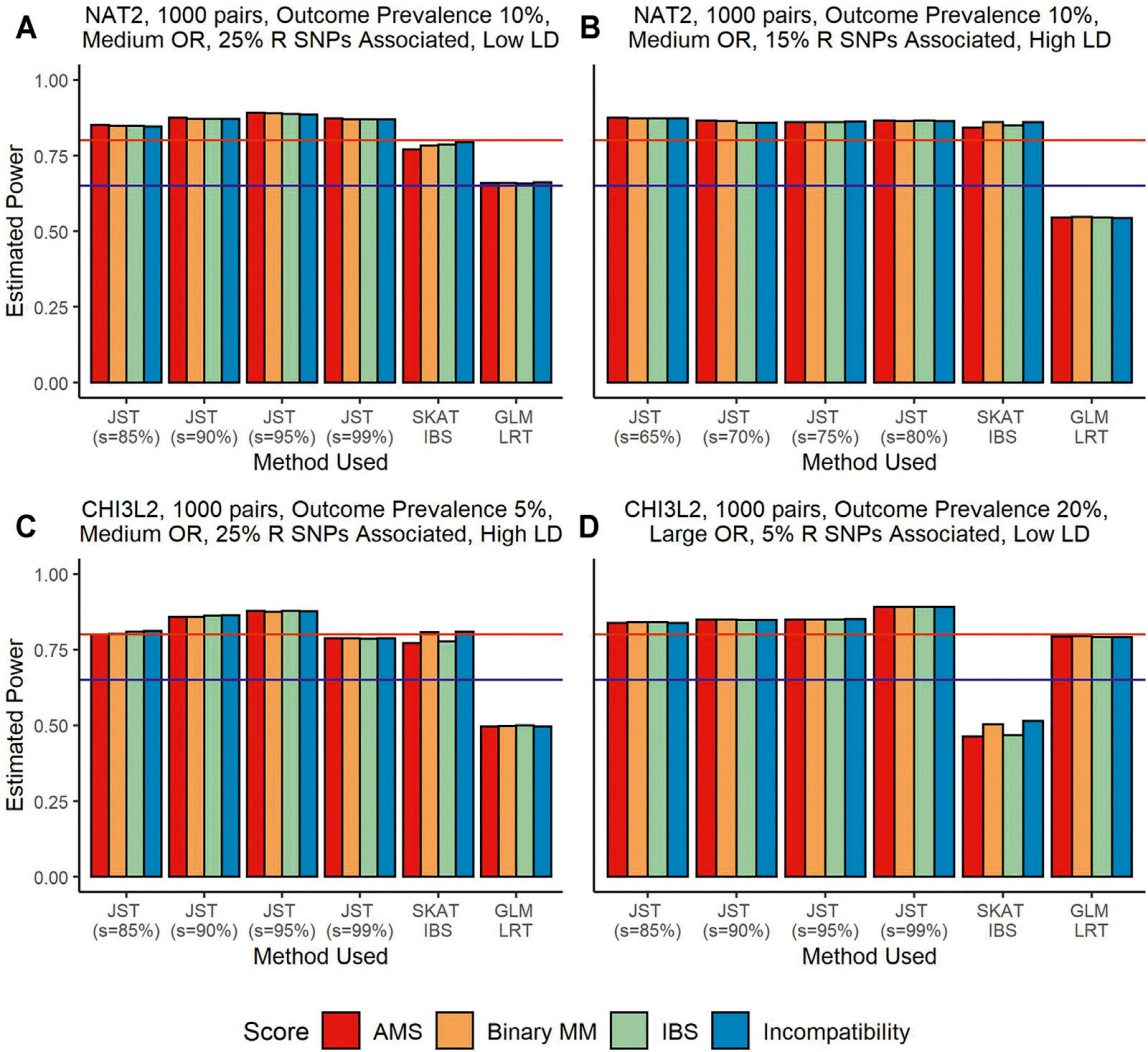
Power estimates from simulations using the genes *NAT2* (Panels **(A,B)**) and *CHI3L2* (Panels **(C,D)**) with 1,000 pairs of donors and recipients under the scenario that recipient genotype SNPs were associated with outcome. Panels **(A,B)** show an outcome prevalence of 10%. Panel **(C)** has an outcome prevalence of 5% and Panel **(D)**, 20%. Panels **(A–C)** show results for a medium OR (1.25) and Panel **(D)** for a large OR (2.00). Panels **(A, C)** show results when 25% of recipient SNPs are associated, while 15% are associated in Panel **(B)** and only 5% in Panel **(D)**. In panels **(A, D)**, SNPs are in low LD while in panels **(B, C)** they are in high LD. The four colored bars represent which gene-based score was fit in the model, with red corresponding to the allogenomics mismatch score (AMS), orange to binary mismatch score, green to identity-by-state (IBS) score, and blue to Incompatibility score. From left to right in panels **(A, C,D)**, the method used for model fitting was the joint score test (JST), with s values of 85, 90, 95, and 99% of variance explained by the principal components (PCs), the sequence kernel association test (SKAT) with the unweighted IBS kernel, and a generalized linear model (GLM) likelihood ratio test (LRT). Panel **(B)** shows results for JST with s values of 65–80%. The y-axis shows estimated power from 0 to 100%. The horizontal blue line corresponds to 65% power and the horizontal red line corresponds to 80% power.

Figure 1 shows results from power analyses where the recipient genotype SNPs are associated with outcome, but the gene-based matching score is not associated. Under this scenario, SKAT using the IBS kernel performs better than SKAT using the linear kernel, so we use the IBS kernel results for our comparisons. The GLM LRT tends to have the lowest power, ranging from around 50% (Figures 1B,C) to around 80% (Figure 1D). It is probable that the power levels in panel D are being artificially inflated due to inflation of type I error rate. The power difference between the proposed JST and SKAT using the unweighted IBS kernel varies. When the R genotype SNPs associated with outcome are in low LD, JST tends to have higher power than SKAT, as seen in the Figure 1A where JST with *s* = 95% reaches close to 90% power but SKAT does not reach 80% power. When the SNPs associated with the outcome are in high LD, SKAT and the JST method can have similar power levels. This can be seen in the Figure 1B where both SKAT and JST with *s* = 65–80% reach around 85% power. When the size of the gene is increased, as in panels C and D, we see that JST tends to have higher power than SKAT in both scenarios. Additionally, when a continuous outcome is examined, JST with any value of *s* tends to have much higher power than SKAT using either kernel (Supplementary Figure S2).

For this scenario, there is not a singular *s* value that always results in the highest power. Figures 1A,D both show that JST has the highest power when *s* corresponds to 95% variance explained. Panel B shows that under different circumstances, retaining a smaller number of principal components may result in higher power, while the opposite is seen to be true for Figure 1D. We note that power levels for JST in all panels are around 80%, so changing the number of principal components retained does not seem to drastically affect the overall power of the method.

#### 3.1.3 Power—Donor/Recipient Gene-Based Matching Score Associated

**FIGURE 2:**
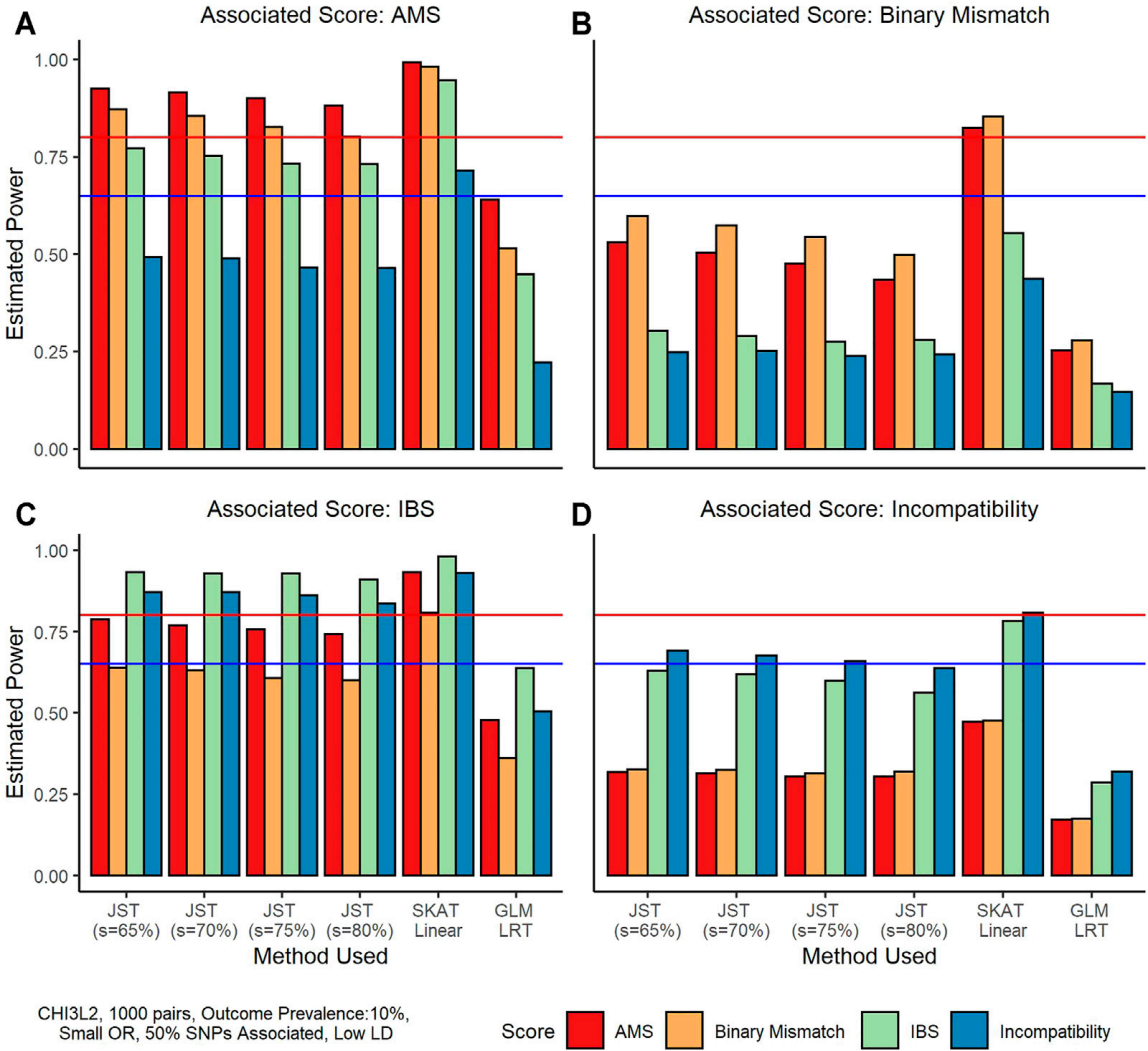
Power estimates from simulations using the gene *CHI3L2* and 1,000 pairs of donors and recipients under the scenario that the gene-based score was associated with outcome. All plots shown are for a binary outcome with prevalence 10%. A small odds ratio (1.25) was used for phenotype generation. For these simulations, 50% of SNPs in the gene score were associated with the outcome, and these SNPs were in low LD. From left to right, and top to bottom the true associated gene-score is the allogenomics mismatch score (AMS), the Binary Mismatch, the identity-by-state (IBS), and the Incompatibility score. The four colors represent which score was used to fit the model, where red is the AMS, yellow is the Binary Mismatch score, green is the IBS, and blue is the Incompatibility score. In each plot, the x-axis corresponds to the method used, where from left to right methods are joint score test (JST) with s values of 65, 70, 75, and 80% of variance explained by the principal components used, the sequence kernel association test (SKAT) with unweighted linear kernel, and a generalized linear model (GLM) likelihood ratio test (LRT). The y-axis shows estimated power from 0 to 100%. The horizontal blue line corresponds to 65% power and the horizontal red line corresponds to 80% power.

Figure 2 shows an example of results on power when the D/R gene-based matching score was associated with outcome and recipient genotype was not associated. Under this scenario, SKAT performance is slightly better using the linear kernel versus the IBS kernel, so we use the linear kernel results in our comparison. We can see that overall, SKAT has the highest power under these simulation conditions, with power levels reaching at least 80% for all four associated scores. The proposed JST method has the second highest power ranging from around 60% when the binary mismatch score is associated to around 90% when the IBS score is associated. The GLM LRT has the lowest power, ranging from around 25% to almost 65%. We note that JST tends to have the highest power when the percentage of variance explained by the *s* PCs is the smallest (65%). When the IBS gene-based score or the AMS is truly associated (Figures 2A,C), power tends to be higher overall than when the binary scores are associated, with all *s* values leading to over 80% power. The Binary mismatch score being truly associated (Figure 2B) tends to have the lowest overall power of the four scores, with four scenarios in which power does not reach 65%. Similar results are seen when outcome is continuous (Supplementary Figure S3).

### 3.2 Data Analysis Results

Table 2 shows the top five genes from analysis of the combined GEN03 and DeKAF data sets using JST with an *s* value of 85% and the top five genes from SKAT analysis of the combined data sets, using an unweighted IBS kernel. Gene ranking is based on the smallest *p*-value for all four score models. *p*-values for the four different models are relatively similar for each of the five genes. The number of genotyped SNPs in the genes ranges from 3 to 15 for the JST results and from 3 to 104 in the SKAT results. Only one of the top genes found using SKAT analyses was also found in the top five for JST analysis. All five of the genes from JST analysis are significant using a FDR cutoff of less than 0.10, while none of the genes found by SKAT are significant using this method.

**TABLE 2:** Results of JST and SKAT analysis of combined GEN03 and DeKAF data sets. The top 5 genes sorted by *p*-value from fitting a model with the any of the four gene-based score are shown. JST was calculated using *p* = 85% of variance explained. SKAT was run using the IBS kernel. Adj. *p*-value: *p*-value adjusted for multiple comparisons using FDR, IBS: identity-by-state, Incomp: incompatibility, AMS: allogenomics mismatch score.

Gene ID	IBS Score	*p*-value	Adj. *p*-value	Incomp. Score	*p*-value	Adj. *p*-value	AMS Score	*p*-value	Adj. *p*-value	Binary Mismatch Score	*p*-value	Adj. *p*-value
JST Analysis Results												
*IFNA5*	22.62	4.86E-05	0.25	27.2	5.33E-06	0.09	30.29	1.20E-06	0.05	30.29	1.20E-06	0.05
*AC002511.1 *	22.66	1.20E-05	0.09	26.77	1.54E-06	0.05	17.92	1.28E-04	0.38	23	1.01E-05	0.09
*NTRK3-AS1*	25.15	1.44E-05	0.09	27.58	4.44E-06	0.09	16.29	9.90E-04	0.65	17.1	6.75E-04	0.56
*Z98752.3*	28.4	1.04E-05	0.09	27.94	1.28E-05	0.09	28.16	1.16E-05	0.09	27.81	1.36E-05	0.09
*SGK2*	27.66	1.46E-05	0.09	27.59	1.51E-05	0.09	27.6	1.50E-05	0.09	27.58	1.51E-05	0.09
**SKAT Analysis Results**												
*AC002511.1*	150.97	6.49E-06	0.32	151.46	5.28E-06	0.32	86.24	7.36E-05	0.80	87.86	5.71E-05	0.80
*AC117569.1*	79.7	1.29E-03	0.82	72.84	1.67E-03	0.82	109.16	9.65E-05	0.80	111.07	3.64E-05	0.80
*AC104041.1*	42.43	3.79E-04	0.80	35.16	1.26E-03	0.82	45.09	5.40E-05	0.80	38.05	1.71E-04	0.80
*LINC01968*	92.71	8.58E-05	0.80	84.02	8.07E-05	0.80	66.94	1.06E-03	0.82	65.23	4.97E-04	0.80
*COG4*	19.18	2.11E-04	0.80	14.35	1.80E-03	0.82	19.87	8.95E-05	0.80	16.97	2.52E-04	0.80

## 4 Discussion

We propose a multi-marker test statistic designed for use with paired genetic data in transplantation. The joint score test, or JST, can be used for testing whether specific gene regions are associated with transplant outcomes, either through the recipient genotype or D/R genetic matching. Compared to other statistical tests in this setting, the JST has the potential for increased power and reproducibility as compared to single SNP tests, as well as increased interpretability as compared to multi-SNP methods that sum across large regions of the genome.

The Eigen decomposition of the recipient genotype covariance matrix allows for a potential increase in power for JST as compared to a standard GLM likelihood ratio test. By transforming the covariance matrix into its principal components, we are able to select only those components that are most likely to be associated and discard the remaining components. We chose to keep principal components with large eigenvalues in accordance with previous theoretical and empirical work by Liu et al. (2020) in order to have the greatest power to detect genetic associations.

Our simulations showed that JST is a competitive method as compared to standard GLM, and SKAT. Type I error rates were conserved for both JST and SKAT but were inflated for GLM. This inflation could be due to the larger number of covariates being fit in this model. In power simulations, JST tended to outperform SKAT when recipient genotype SNPs, in low LD with all other SNPs in the region, were truly associated with outcome. When the recipient SNPs were in high LD, SKAT and JST performed similarly for the smallest gene but increasing the gene size while keeping the percentage of associated SNPs constant led to JST surpassing SKAT in power. These findings agree with those found by Liu et al. (2020) where the SKAT method performed closer to use of the first principal component when LD between SNPs increased, due to the increase of the first eigenvalue weighting the first PC higher in the SKAT test statistic. When gene-based score was associated with outcome, SKAT tended to have higher or similar power to JST depending on which gene-based score was used in modeling. When the AMS and IBS score were associated with the outcome, they tended to have higher power than when the Binary Mismatch score and Incompatibility score were associated. Additionally, when the Binary Mismatch score or Incompatibility score were associated with outcome, the AMS or IBS score respectively maintained relatively high power. Following these observations, it is recommended that the AMS or IBS score be used for testing. Ideally, the choice of score should be determined based on prior knowledge of the genetic mechanism.

JST and SKAT behaved similarly in some of the simulation scenarios but the construction of these two test statistics is different. Using the linear kernel, the SKAT statistic is equivalent to summing the squared score statistics of the genetic data (Wu et al., 2011). Under this scenario, SKAT is similar to JST but does not involve the variance between the marginal score functions. The main difference between the two approaches lies in different modeling approaches. SKAT is also based on a generalized linear model, but the log-odds ratio parameters for both the recipient genotypes and gene score are further modeled as following a mean zero distribution. Different ways of specifying variance in such mean zero distribution correspond to different kernel functions chosen for SKAT analysis (Sun et al., 2013). It is not straightforward to derive general insights on which method may be more powerful, but it does seem that their power would differ at least according to the true underlying genetic model and the LD structures of the genetic variants. We recommend both methods be applied for analysis with adjustment to multiple testing.

To the best of our knowledge, SKAT has not previously been evaluated under the scenario of jointly testing for an association between a set of SNPs and a gene-based score and has not been utilized with paired transplant genetics data. Our evaluation of SKAT under these circumstances found that the method works well and is robust. We determined that the choice of kernel often impacted power levels, however, such as when the SKAT method using the linear kernel had minimal power as compared to the SKAT method using the IBS kernel under the scenario where R genotype SNPs were associated with outcome. Investigation into this phenomenon found that scaling the gene-based scores from between 0 and 1, before running SKAT with a linear kernel leads to an improvement in power (Supplementary Figure S4). The IBS kernel had relatively high power under both power scenarios, with power levels only about 5% less than those of the linear kernel when gene-based score was associated, so use of the IBS kernel may be preferred when the true underlying association is unknown.

Based on the simulation studies, there is no clear value of *s* that leads to the highest power in all scenarios. When gene-based score was associated with the outcome, the smallest *s* value we considered tended to have the highest power, but power tended to be similar for *s* values corresponding to between 65 and 80% variance explained by the PCs. When recipient genotype SNPs were associated with the outcome, changing the value of *s* did not tend to drastically change the power levels. Since the true association mechanism is unknown for real data analysis, it may be beneficial to run models using a few different *s* values, although this will increase the number of tests. An alternative is to choose a middling value of *s*, around 80 or 85%, which tends to have high power under either scenario of association.

Our analysis of kidney transplant data found five genes to be statistically significant after accounting for multiple comparisons at FDR<0.10. These include genes that could plausibly lead to AR after kidney transplant. Three of the five genes, *IFNA5, Z98752.3,* and *SGK2*, have been found to be associated with the immune system or specific types of immune cells which could attack a transplanted kidney if the graft is recognized as non-self (Kichaev et al., 2019; Chen et al., 2020). *IFNA5*, for example, is known to be involved in differentiation and proliferation of B and T cells, as well as being involved in the adaptive immune response, which involves the creation of antibodies that may attack a donor organ (Huntley et al., 2015). In previous GWAS analyses, SNPs located in *SGK2* were found to be associated with leukocyte and monocyte counts which are directly associated with immune response (Kichaev et al., 2019; Chen et al., 2020). Replication of these results will be needed to verify the significance of these findings.

The JST method does have some limitations. Transplant data analysis was limited to data from paired kidney transplants. The method can be applied to other organ data as well, as long as genotype data is available for both the donor and the recipient. For our simulation studies, we only focused on unrelated D/R pairs, but it is possible that the degree of relatedness between a donor and a recipient may impact whether the recipient experiences acute rejection. We were able to look at related versus non-related pairs in our combined kidney data sets and found that these two groups had no overlap in their top five potentially associated genes. Based on these results, we believe it is important to account for relatedness between a D/R pair in analyses. We restricted our analyses to only include common SNPs, but rare variants can be used in JST analyses. If there is interest in the association of rare variants, it is possible to construct a weighted version of the distance function as,

$$Z_{i}=\frac{\sum_{j=1}^{m}w_{j}D\left(X_{ij}^{D}\;,\;X_{ij}^{R}\;\right)}{\sum_{j=1}^{m}w_{j}}$$

Then a simple weighting option that will help upweight rarer minor allele frequencies is the use of wj=1MAFj (Kwee et al., 2008). Additionally, the JST method gives results based on the joint null hypothesis of either recipient genotype SNP effects and/or gene-based score effect but cannot specify which effect is driving the results. Work is ongoing to determine a testing method for gene-based score effect that can account for any recipient genotype SNP effect simultaneously.

In summary, the JST is a powerful method that can be used for the analysis of paired genetic data. Use of this method could lead to the discovery of gene regions potentially important to transplant outcome, which could be further studied to try and determine the biological mechanisms behind acute rejection.

## Data Availability

The original contributions presented in the study are included in the article/Supplementary Material, further inquiries can be directed to the corresponding author.
