# Supplementary material for: A Multi-Marker Test for Analyzing Paired Genetic Data in Transplantation
Source: Front Genet. 2021 Oct 13;12:745773. doi: 10.3389/fgene.2021.745773 (PMC8548646; doi:10.3389/fgene.2021.745773)
Supplement: Supplementary file 1 [file DataSheet1.docx]

Supplementary Material

# Derivation of the JST Statistic

Let $\boldsymbol{W}_{\boldsymbol{i}}=\left( W_{i1},...,W_{iK} \right)$ denote the vector of covariates for adjustment, which may include PCs for capturing population substructure. Our JST test statistic was based on the following logistic regression model,

$$logit Pr\left( Y_{i}=1 | \boldsymbol{X}_{\boldsymbol{i}}\boldsymbol{,}Z_{i} \right)= \alpha_{0}+ \sum_{k=1}^{K} W_{ik}\alpha_{k}+ \sum_{j=1}^{m} X_{ij}\beta_{j}+ Z_{i}\gamma.$$

We would like to test the null hypotheses $H_{0}:\boldsymbol{\beta}=0$ and $\gamma=0$. Let $\hat{\alpha}_{0}$ and $\hat{\boldsymbol{\alpha}}=(\hat{\alpha}_{1},\ldots,\hat{\alpha}_{k})'$denote estimates of $\alpha_{0}$ and $\boldsymbol{\alpha}=(\alpha_{1},\ldots,\alpha_{k})'$ under the null, which are obtained by regressing $\boldsymbol{Y}=(Y_{1},\ldots,Y_{n})'$ only on the covariates ${\boldsymbol{W}=(\boldsymbol{W}}_{\boldsymbol{1}},\ldots,\boldsymbol{W}_{\boldsymbol{n}})'$ but not ***X*** or *Z*. Let $\hat{\boldsymbol{p}}={logit}^{-1}\left( \hat{\alpha}_{0}+\boldsymbol{W}\hat{\boldsymbol{\alpha}} \right)=(\hat{p}_{1},\ldots,\hat{p}_{n})'$ denote the predicted probability of $Y$under $H_{0}$.

Define $P_{1}(\boldsymbol{W})$ and $P_{0}(\boldsymbol{W})$ as

$P_{1}\left( \boldsymbol{W}_{\boldsymbol{i}} \right)= \frac{e^{\alpha_{0}+ \boldsymbol{W}_{\boldsymbol{i}}\boldsymbol{\alpha}}}{1+e^{\alpha_{0}+ \boldsymbol{W}_{\boldsymbol{i}}\boldsymbol{\alpha}}}$ and $P_{0}\left( \boldsymbol{W}_{\boldsymbol{i}} \right){=1- P}_{1}\left( \boldsymbol{W}_{\boldsymbol{i}} \right)= \frac{1}{1+e^{\alpha_{0}+ \boldsymbol{W}_{\boldsymbol{i}}\boldsymbol{\alpha}}}$.

Then the marginal likelihood score function for $\boldsymbol{\beta}=(\beta_{1},\ldots{,\beta}_{m})'$ under the null can be shown equal to

$U^{R}=\left( U_{1}^{R},\ldots,U_{m}^{R} \right)^{'}= \sum_{i=1}^{n} \boldsymbol{X}_{\boldsymbol{i}}'(Y_{i}- \frac{e^{\hat{\alpha}_{0}+ \boldsymbol{W}_{\boldsymbol{i}}\hat{\boldsymbol{\alpha}}}}{1+e^{\hat{\alpha}_{0}+ \boldsymbol{W}_{\boldsymbol{i}}\hat{\boldsymbol{\alpha}}}})$,

and the marginal likelihood score function for $\gamma$ under the null can be shown equal to

$U^{S}= \sum_{i=1}^{n} Z_{i}(Y_{i}- \frac{e^{\hat{\alpha}_{0}+ \boldsymbol{W}_{\boldsymbol{i}}\hat{\boldsymbol{\alpha}}}}{1+e^{\hat{\alpha}_{0}+ \boldsymbol{W}_{\boldsymbol{i}}\hat{\boldsymbol{\alpha}}}})$,

By standard Taylor series expansion, these can be further written as

$U^{R}=\sum_{i=1}^{n} \boldsymbol{X}_{\boldsymbol{i}}'(Y_{i}- \frac{e^{\alpha_{0}+ \boldsymbol{W}_{\boldsymbol{i}}\boldsymbol{\alpha}}}{1+e^{\alpha_{0}+ \boldsymbol{W}_{\boldsymbol{i}}\boldsymbol{\alpha}}})- \sum_{i=1}^{n} \boldsymbol{X}_{\boldsymbol{i}}\boldsymbol{'}\left( 1,\boldsymbol{W}_{\boldsymbol{i}} \right)P_{0}\left( \boldsymbol{W}_{\boldsymbol{i}} \right)P_{1}(\boldsymbol{W}_{\boldsymbol{i}})\binom{\hat{\alpha}_{0}- \alpha_{0}}{\hat{\boldsymbol{\alpha}}\boldsymbol{- \alpha}}$

and

$U^{S}=\sum_{i=1}^{n} Z_{i}(Y_{i}- \frac{e^{\alpha_{0}+ \boldsymbol{W}_{\boldsymbol{i}}\boldsymbol{\alpha}}}{1+e^{\alpha_{0}+ \boldsymbol{W}_{\boldsymbol{i}}\boldsymbol{\alpha}}})- \sum_{i=1}^{n} Z_{i}\left( 1,\boldsymbol{W}_{\boldsymbol{i}} \right)P_{0}\left( \boldsymbol{W}_{\boldsymbol{i}} \right)P_{1}(\boldsymbol{W}_{\boldsymbol{i}})\binom{\hat{\alpha}_{0}- \alpha_{0}}{\hat{\boldsymbol{\alpha}}\boldsymbol{- \alpha}}$.

Note that $\hat{\alpha}_{0}$ and $\hat{\boldsymbol{\alpha}}=(\hat{\alpha}_{1},\ldots,\hat{\alpha}_{k})'$ are solutions to the following score equation:

$$0= \sum_{i=1}^{n} \binom{1}{\boldsymbol{W}_{\boldsymbol{i}}\boldsymbol{'}}\left( Y_{i}- \frac{e^{\hat{\alpha}_{0}+ \boldsymbol{W}_{\boldsymbol{i}}\hat{\boldsymbol{\alpha}}}}{1+e^{\hat{\alpha}_{0}+ \boldsymbol{W}_{\boldsymbol{i}}\hat{\boldsymbol{\alpha}}}} \right).$$

Standard Taylor series expansion leads to the following,

$0= \sum_{i=1}^{n} \binom{1}{\boldsymbol{W}_{\boldsymbol{i}}\boldsymbol{'}}\left( Y_{i}- \frac{e^{\alpha_{0}+ \boldsymbol{W}_{\boldsymbol{i}}\boldsymbol{\alpha}}}{1+e^{\alpha_{0}+ \boldsymbol{W}_{\boldsymbol{i}}\boldsymbol{\alpha}}} \right)- \sum_{i=1}^{n} \binom{1}{\boldsymbol{W}_{\boldsymbol{i}}\boldsymbol{'}}(1,\boldsymbol{W}_{\boldsymbol{i}})P_{0}\left( \boldsymbol{W}_{\boldsymbol{i}} \right)P_{1}(\boldsymbol{W}_{\boldsymbol{i}})\binom{\hat{\alpha}_{0}- \alpha_{0}}{\hat{\boldsymbol{\alpha}}\boldsymbol{- \alpha}}$.

After rearranging the terms, we obtain

$\binom{\hat{\alpha}_{0}- \alpha_{0}}{\hat{\boldsymbol{\alpha}}\boldsymbol{- \alpha}}=\left\{ \sum_{i=1}^{n} \binom{1}{\boldsymbol{W}_{\boldsymbol{i}}\boldsymbol{'}}(1,\boldsymbol{W}_{\boldsymbol{i}})P_{0}\left( \boldsymbol{W}_{\boldsymbol{i}} \right)P_{1}(\boldsymbol{W}_{\boldsymbol{i}}) \right\}^{-1}\left\{ \sum_{i=1}^{n} \binom{1}{\boldsymbol{W}_{\boldsymbol{i}}\boldsymbol{'}}\left( Y_{i}- \frac{e^{\alpha_{0}+ \boldsymbol{W}_{\boldsymbol{i}}\boldsymbol{\alpha}}}{1+e^{\alpha_{0}+ \boldsymbol{W}_{\boldsymbol{i}}\boldsymbol{\alpha}}} \right) \right\}$.

Therefore, $U^{R}$ can be written as

$$U^{R}= \sum_{i=1}^{n} \boldsymbol{[X}_{\boldsymbol{i}}\boldsymbol{'}-\left\{ \sum_{i=1}^{n} \boldsymbol{X}_{\boldsymbol{i}}'\left( 1,\boldsymbol{W}_{\boldsymbol{i}} \right)P_{0}\left( \boldsymbol{W}_{\boldsymbol{i}} \right)P_{1}\left( \boldsymbol{W}_{\boldsymbol{i}} \right) \right\}\left\{ \sum_{i=1}^{n} \binom{1}{\boldsymbol{W}_{\boldsymbol{i}}\boldsymbol{'}}\left( 1,\boldsymbol{W}_{\boldsymbol{i}} \right)P_{0}\left( \boldsymbol{W}_{\boldsymbol{i}} \right)P_{1}\left( \boldsymbol{W}_{\boldsymbol{i}} \right) \right\}^{-1}\binom{1}{\boldsymbol{W}_{\boldsymbol{i}}\boldsymbol{'}}]\left( Y_{i}- \frac{e^{\alpha_{0}+ \boldsymbol{W}_{\boldsymbol{i}}\boldsymbol{\alpha}}}{1+e^{\alpha_{0}+ \boldsymbol{W}_{\boldsymbol{i}}\boldsymbol{\alpha}}} \right).$$

Similarly, $U^{S}$ can be written as

$$U^{S}=\sum_{i=1}^{n} \left[ Z_{i}\boldsymbol{-} \left\{ \sum_{i=1}^{n} Z_{i}\left( 1,\boldsymbol{W}_{\boldsymbol{i}} \right)P_{0}\left( \boldsymbol{W}_{\boldsymbol{i}} \right)P_{1}\left( \boldsymbol{W}_{\boldsymbol{i}} \right) \right\}\left\{ \sum_{i=1}^{n} \binom{1}{\boldsymbol{W}_{\boldsymbol{i}}\boldsymbol{'}}\left( 1,\boldsymbol{W}_{\boldsymbol{i}} \right)P_{0}\left( \boldsymbol{W}_{\boldsymbol{i}} \right)P_{1}\left( \boldsymbol{W}_{\boldsymbol{i}} \right) \right\}^{-1}\binom{1}{\boldsymbol{W}_{\boldsymbol{i}}} \right](Y_{i}- \frac{e^{\alpha_{0}+ \boldsymbol{W}_{\boldsymbol{i}}\boldsymbol{\alpha}}}{1+e^{\alpha_{0}+ \boldsymbol{W}_{\boldsymbol{i}}\boldsymbol{\alpha}}}).$$

Further, let $\hat{X}_{ij}$ denote the fitted value for the *j*^th^ recipient genotype SNP for the *i*^th^ recipient from the weighted linear regression model $X_{ij}= \theta_{0}+ \sum_{k=1}^{K} W_{ik}\theta_{k}+\varepsilon_{X}$ and let $\hat{Z}_{i}$ denote the fitted value for the gene-based genetic matching score of D/R pair *i* from a weighted linear regression model $Z_{i}= \tau_{0}+$ $\sum_{k=1}^{K} W_{ik}\tau_{k}+ \varepsilon_{Z}$. In both cases, the weights are $\hat{p}_{1}\left( \boldsymbol{W}_{\boldsymbol{i}} \right)\{1- \hat{p}_{1}\left( \boldsymbol{W}_{\boldsymbol{i}} \right)\}$ for recipient *i* or D/R pair *i*. Following standard weighted ordinary least squares procedures, we calculate that $\binom{\hat{\theta_{0}}}{\hat{\theta}}= \left\{ \sum_{i=1}^{n} \binom{1}{\boldsymbol{W}_{\boldsymbol{i}}\boldsymbol{'}}\left( 1,\boldsymbol{W}_{\boldsymbol{i}} \right)P_{0}\left( \boldsymbol{W}_{\boldsymbol{i}} \right)P_{1}\left( \boldsymbol{W}_{\boldsymbol{i}} \right) \right\}^{-1}\left\{ \sum_{i=1}^{n} \boldsymbol{X}_{\boldsymbol{i}}'\left( 1,\boldsymbol{W}_{\boldsymbol{i}} \right)P_{0}\left( \boldsymbol{W}_{\boldsymbol{i}} \right)P_{1}\left( \boldsymbol{W}_{\boldsymbol{i}} \right) \right\}$and $\binom{\hat{\tau_{0}}}{\hat{\tau}}= \left\{ \sum_{i=1}^{n} \binom{1}{\boldsymbol{W}_{\boldsymbol{i}}\boldsymbol{'}}\left( 1,\boldsymbol{W}_{\boldsymbol{i}} \right)P_{0}\left( \boldsymbol{W}_{\boldsymbol{i}} \right)P_{1}\left( \boldsymbol{W}_{\boldsymbol{i}} \right) \right\}^{-1}\left\{ \sum_{i=1}^{n} \boldsymbol{Z}_{\boldsymbol{i}}\left( 1,\boldsymbol{W}_{\boldsymbol{i}} \right)P_{0}\left( \boldsymbol{W}_{\boldsymbol{i}} \right)P_{1}\left( \boldsymbol{W}_{\boldsymbol{i}} \right) \right\}.$ This leads to $\hat{X}_{ij}= \left\{ \sum_{i=1}^{n} \boldsymbol{X}_{\boldsymbol{i}}'\left( 1,\boldsymbol{W}_{\boldsymbol{i}} \right)P_{0}\left( \boldsymbol{W}_{\boldsymbol{i}} \right)P_{1}\left( \boldsymbol{W}_{\boldsymbol{i}} \right) \right\}\left\{ \sum_{i=1}^{n} \binom{1}{\boldsymbol{W}_{\boldsymbol{i}}\boldsymbol{'}}\left( 1,\boldsymbol{W}_{\boldsymbol{i}} \right)P_{0}\left( \boldsymbol{W}_{\boldsymbol{i}} \right)P_{1}\left( \boldsymbol{W}_{\boldsymbol{i}} \right) \right\}^{-1}\binom{1}{\boldsymbol{W}_{\boldsymbol{i}}\boldsymbol{'}}$ and $\hat{Z}_{i}= \left\{ \sum_{i=1}^{n} Z_{i}\left( 1,\boldsymbol{W}_{\boldsymbol{i}} \right)P_{0}\left( \boldsymbol{W}_{\boldsymbol{i}} \right)P_{1}\left( \boldsymbol{W}_{\boldsymbol{i}} \right) \right\}\left\{ \sum_{i=1}^{n} \binom{1}{\boldsymbol{W}_{\boldsymbol{i}}\boldsymbol{'}}\left( 1,\boldsymbol{W}_{\boldsymbol{i}} \right)P_{0}\left( \boldsymbol{W}_{\boldsymbol{i}} \right)P_{1}\left( \boldsymbol{W}_{\boldsymbol{i}} \right) \right\}^{-1}\binom{1}{\boldsymbol{W}_{\boldsymbol{i}}}.$

It is easy to see that the *j*^th^ component of $U^{R}$, $j=(1,\ldots,m)$ can be re-written as

$$U_{j}^{R}= \sum_{i=1}^{n} X_{ij}\left( Y_{i}-\hat{p}_{1} \right)= \sum_{i=1}^{n} (X_{ij}-\hat{X}_{ij})(Y_{i}-p_{1})$$

and $U^{S}$ can be written as

$U^{S}= \sum_{i=1}^{n} Z_{i}\left( Y_{i}-\hat{p}_{1} \right)= \sum_{i=1}^{n} (Z_{i}-\hat{Z}_{i})(Y_{i}-p_{1})$.

If we let $\boldsymbol{B}_{\boldsymbol{i}}=(\boldsymbol{X}_{\boldsymbol{i}},Z_{i})$ and ${\hat{\boldsymbol{B}}}_{i}=\left( {\hat{\boldsymbol{X}}}_{\boldsymbol{i}},\hat{Z}_{i} \right)$ we can write ***U*** in matrix form as $\boldsymbol{U}=\left( \boldsymbol{B}-\hat{\boldsymbol{B}} \right)^{T}\{\boldsymbol{Y}-{\hat{\boldsymbol{p}}}_{\boldsymbol{1}}\}$. The matrix ***V*** can therefore be computed using the formula provided in the text.

Under the null hypothesis, $\boldsymbol{U}\to^{d}\mathcal{N}_{m+1}(\boldsymbol{0},\boldsymbol{V})$ by multivariate central limit theorem (CLT) and Slutsky’s theorem as *N* becomes large, so the Hotelling’s *T*^2^, $n\boldsymbol{U}^{\boldsymbol{T}}{\hat{\boldsymbol{V}}}^{\boldsymbol{-}\boldsymbol{1}}\boldsymbol{U=}n\boldsymbol{U}^{\boldsymbol{T}}{\boldsymbol{(}\hat{\boldsymbol{V}}}^{\boldsymbol{-}\boldsymbol{1/2}}\boldsymbol{)}{\boldsymbol{(}\hat{\boldsymbol{V}}}^{\boldsymbol{-}\boldsymbol{1/2}}\boldsymbol{)U}$, follows a Chi-squared distribution with *m+1* degrees of freedom. After Eigen decomposition, we have $\boldsymbol{U}^{\boldsymbol{P}}\boldsymbol{=(}\boldsymbol{U}^{\boldsymbol{PR}}\boldsymbol{,}\boldsymbol{U}^{\boldsymbol{S}}\boldsymbol{)}$ and $\boldsymbol{U}^{\boldsymbol{P}}\to^{d}\mathcal{N}_{s+1}(\boldsymbol{0},\boldsymbol{\Sigma})$, where $\boldsymbol{\Sigma=}\left[ \begin{matrix} I_{s\times s} & Cov(\boldsymbol{U}^{\boldsymbol{PR}}, U^{S}) \\ Cov(U^{S}, \boldsymbol{U}^{\boldsymbol{PR}}) & Var(U^{S}) \end{matrix} \right]$. Consequently, our final JST statistic asymptotically follows a Chi-squared distribution with *s+1* degrees of freedom under the null.

Following the same steps, we obtain $U_{j}^{R}$ and $U^{S}$ for continuous $Y_{i}$ as

$U_{j}^{R}= \sum_{i=1}^{n} X_{ij}\left( Y_{i}-{{(\hat{\alpha}}_{0}\boldsymbol{+W}}_{\boldsymbol{i}}\hat{\boldsymbol{\alpha}}\boldsymbol{)} \right)= \sum_{i=1}^{n} (X_{ij}-\hat{X}_{ij})(Y_{i}-{{(\alpha}_{0}\boldsymbol{+W}}_{\boldsymbol{i}}\boldsymbol{\alpha}\boldsymbol{)})$

and

$U^{S}= \sum_{i=1}^{n} Z_{i}\left( Y_{i}-{{(\hat{\alpha}}_{0}\boldsymbol{+W}}_{\boldsymbol{i}}\hat{\boldsymbol{\alpha}}\boldsymbol{)} \right)= \sum_{i=1}^{n} (Z_{i}-\hat{Z}_{i})(Y_{i}-{{(\alpha}_{0}\boldsymbol{+W}}_{\boldsymbol{i}}\boldsymbol{\alpha}\boldsymbol{)})$.

# Supplementary Figures and Tables

**Supplementary Table 1** Corresponding number of SNPs that are associated with outcome for genes *NAT2*, *CHI3L2*, and *ASAH1* based on the percent of SNPs associated with outcome.

| Percent of SNPs Associated with Outcome | Number of SNPs Associated with Outcome – *NAT2* | Number of SNPs Associated with Outcome – *CHI3L2* | Number of SNPs Associated with Outcome – *ASAH1* |
| --- | --- | --- | --- |
| 5 | 1 | 2 | 2 |
| 15 | 2 | 5 | 6 |
| 25 | 4 | 8 | 10 |
| 50 | 7 | 17 | 20 |
| 75 | 11 | 25 | 30 |
| 100 | 14 | 33 | 40 |

**Supplementary Table 2** Additional information on the top 5 genes from JST and SKAT analysis shown in Table 2. Chr.: chromosome, bp: base pairs.

| **JST Analysis Results** | | | | |
| --- | --- | --- | --- | --- |
| **Gene ID** | **Chr.** | **Gene Start (bp)** | **Gene End (bp)** | **# SNPs** |
|  |  |  |  |  |
| *IFNA5* | 9 | 21304326 | 21305312 | 6 |
| *AC002511.1* | 19 | 35432957 | 35434642 | 3 |
| *NTRK3-AS1* | 15 | 88252730 | 88271066 | 4 |
| *Z98752.3* | 20 | 43540171 | 43569498 | 15 |
| *SGK2* | 20 | 43558968 | 43588237 | 12 |
| **SKAT Analysis Results** | | | | |
| **Gene ID** | **Chr.** | **Gene Start (bp)** | **Gene End (bp)** | **# SNPs** |
|  |  |  |  |  |
| *AC002511.1* | 19 | 35432957 | 35434642 | 3 |
| *AC117569.1* | 18 | 29518259 | 29548241 | 6 |
| *AC104041.1* | 15 | 81633426 | 82013579 | 104 |
| *LINC01968* | 3 | 194708093 | 194826008 | 62 |
| *COG4* | 16 | 70480568 | 70523560 | 38 |


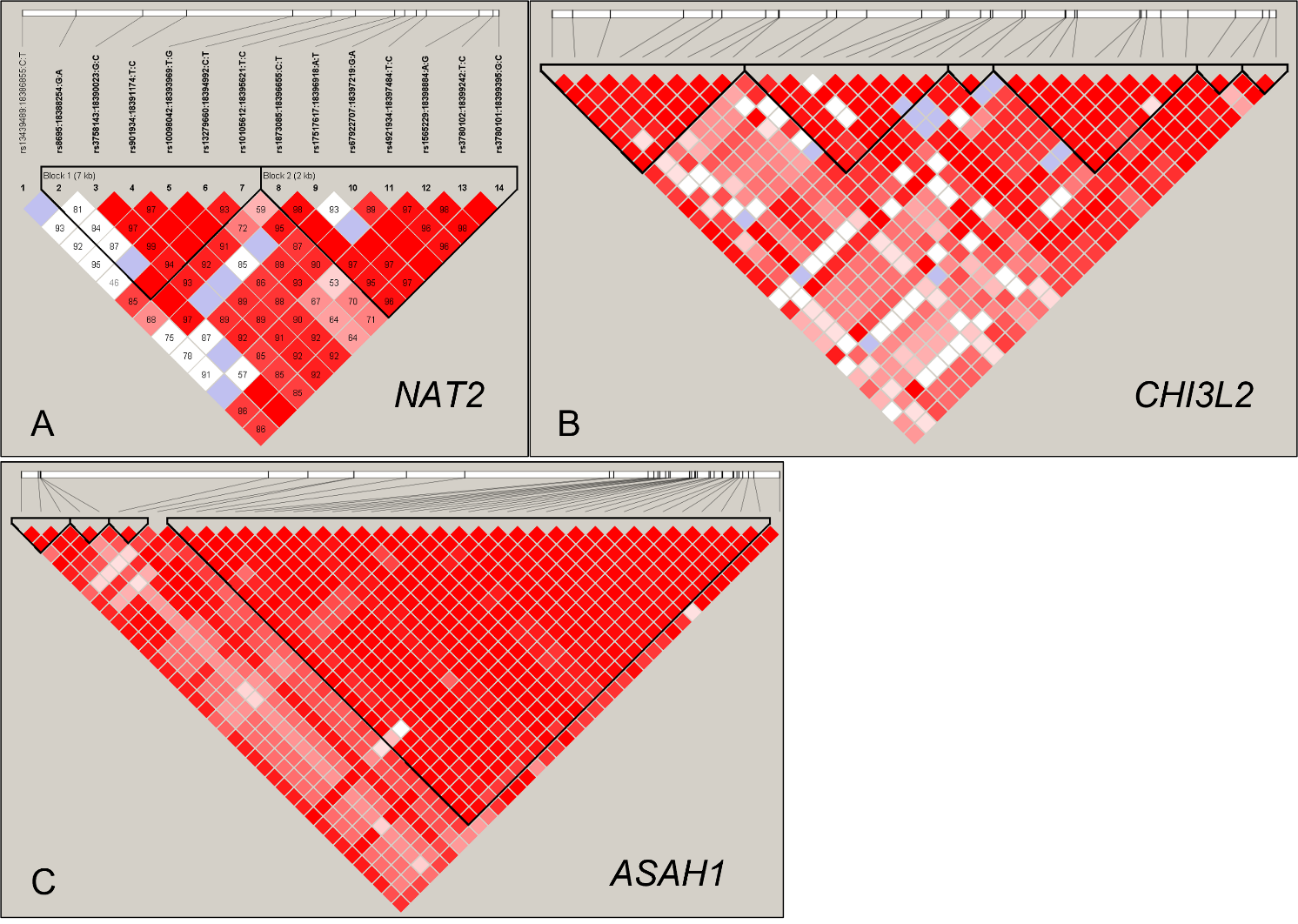


**Supplementary Figure 1.** Haploview plots showing number of SNPs and LD structure for genes *NAT2* (A), *CHI3L2* (B), and *ASAH1* (C) used in simulation analyses.


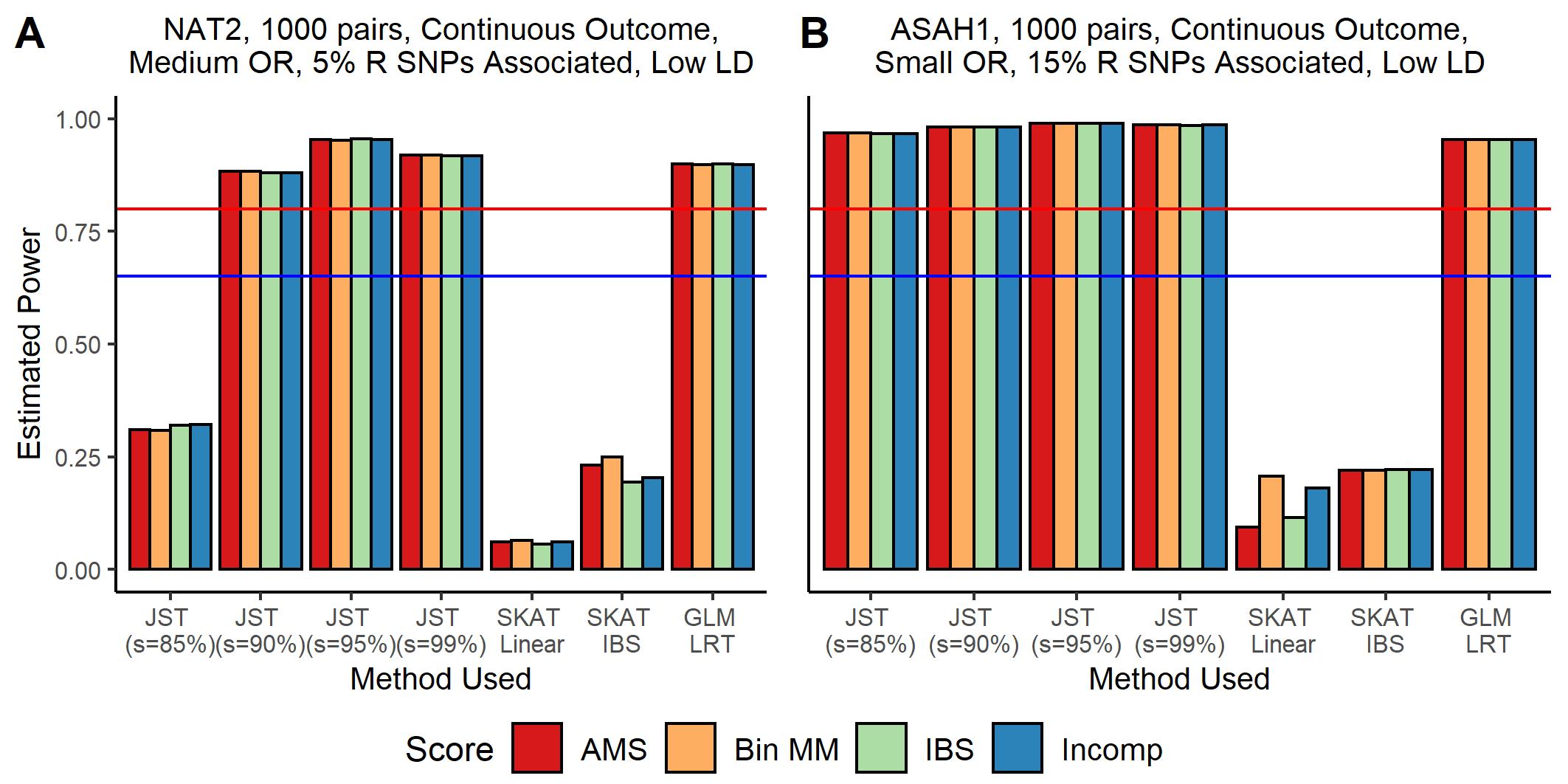


**Supplementary Figure 2.** Power estimates from simulations using 1000 pairs of donors and recipients under the scenario that recipient genotype SNPs were associated with continuous outcome. Panel A shows the scenario when data from *NAT2* was sampled, a medium odds ratio (1.50) was used for phenotype generation and 5% of recipient genotype SNPs were associated with the outcome and were in low LD. Panel B shows the scenario when data from *ASAH1* was sampled, a small odds ratio (1.25) was used for phenotype generation and 15% of recipient genotype SNPs were associated with the outcome and were in low LD. The four colored bars represent which gene-based score was fit in the model, with red corresponding to the allogenomics mismatch score (AMS), orange to binary mismatch score, green to identity-by-state (IBS) score, and blue to Incompatibility score. From left to right in each plot, the method used for model fitting was the joint score test (JST), with s values of 85, 90, 95, and 99% of variance explained by the principal components (PCs), the sequence kernel association test (SKAT) with the unweighted linear and unweighted IBS kernel, and a generalized linear model (GLM) likelihood ratio test (LRT). The y-axis shows estimated power from 0-100%. The horizontal blue line corresponds to 65% power and the horizontal red line corresponds to 80% power.


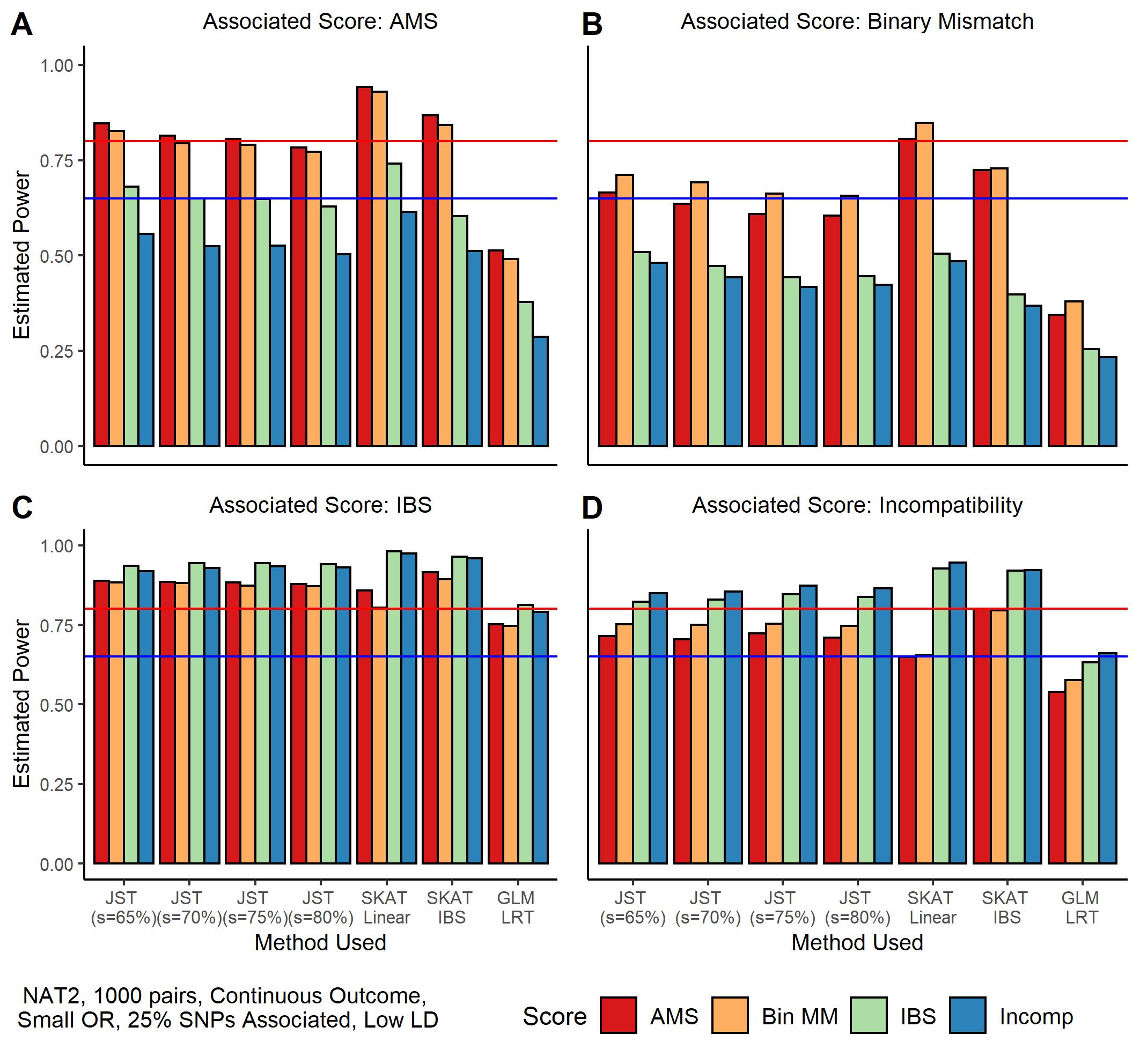


**Supplementary Figure 3.** Power estimates for simulations using the gene *NAT2* and 1000 donor/recipient pairs under the scenario that the gene-based score was associated with continuous outcome. A small odds ratio (1.25) was used for phenotype generation. For these simulations, 25% of SNPs in the gene score were associated with the outcome, and these SNPs were in low LD. From left to right, and top to bottom the true associated gene-score is the allogenomics mismatch score (AMS), the Binary Mismatch, the identity-by-state (IBS), and the Incompatibility score. The four colors represent which score was used to fit the model, where red is the AMS, yellow is the Binary Mismatch score, green is the IBS, and blue is the Incompatibility score. In each plot, the x-axis corresponds to the method used, where from left to right methods are joint score test (JST) with s values of 65, 70, 75, and 80% of variance explained by the principal components used, the sequence kernel association test (SKAT) with unweighted linear kernel, and a generalized linear model (GLM) likelihood ratio test (LRT). The y-axis shows estimated power from 0-100%. The horizontal blue line corresponds to 65% power and the horizontal red line corresponds to 80% power.


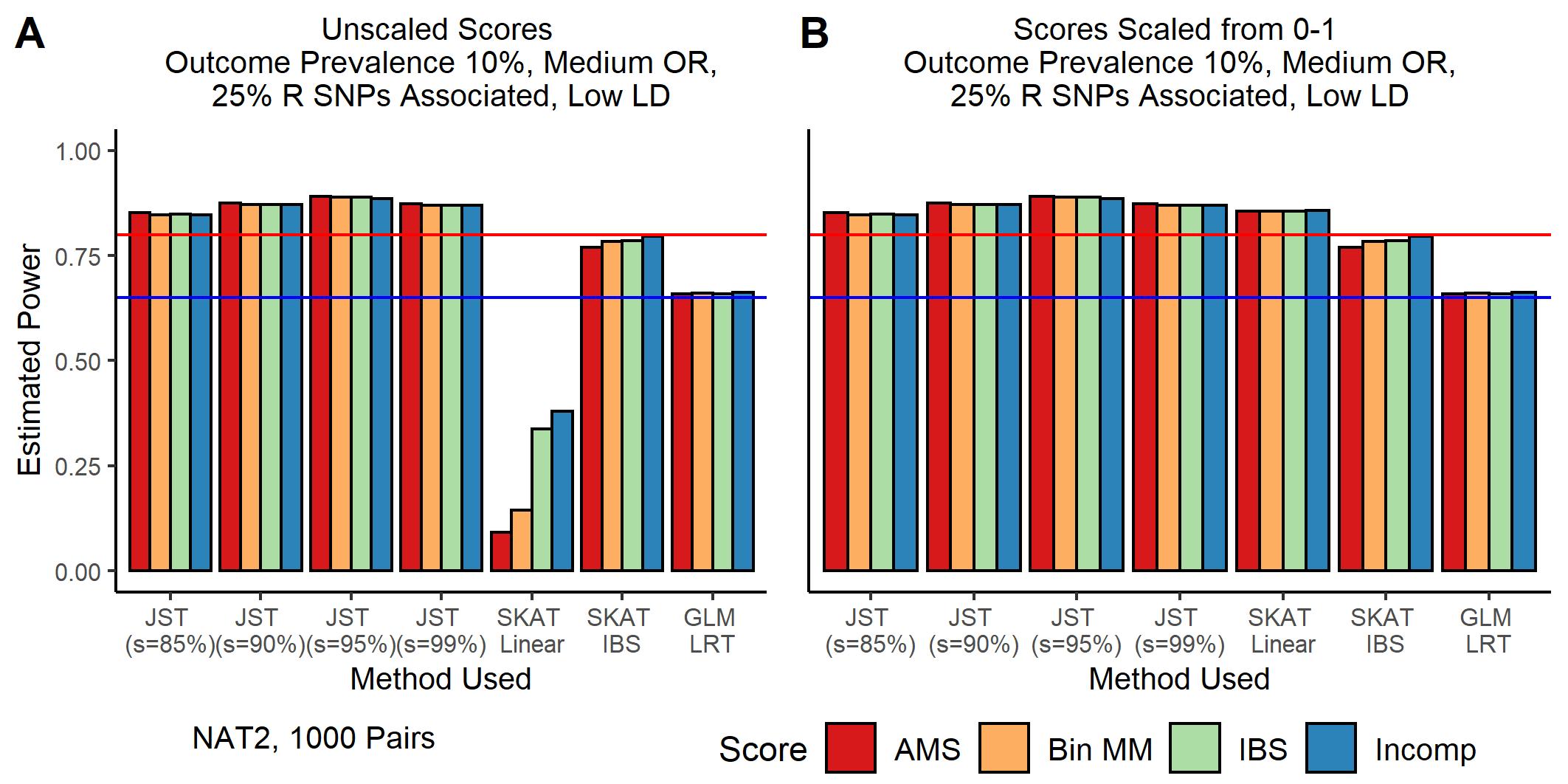


**Supplementary Figure 4.** Power estimates from simulations using data from *NAT2* and 1000 pairs of donors and recipients under the scenario that recipient genotype SNPs were associated with outcome. In both panels, outcome prevalence was 10%, a medium OR (1.50) was used, and 25% of recipient SNPs were associated with outcome and were in low LD. Panel A shows the scenario when SKAT is fit using a linear kernel and the gene-based scores are unscaled. Panel B shows the scenario when SKAT is fit using a linear kernel and gene-based scores are scaled to a range of 0-1. The four colored bars represent which gene-based score was fit in the model, with red corresponding to the allogenomics mismatch score (AMS), orange to binary mismatch score, green to identity-by-state (IBS) score, and blue to Incompatibility score. From left to right in each plot, the method used for model fitting was the joint score test (JST), with s values of 85, 90, 95, and 99% of variance explained by the principal components (PCs), the sequence kernel association test (SKAT) with the unweighted linear and unweighted IBS kernel, and a generalized linear model (GLM) likelihood ratio test (LRT). The y-axis shows estimated power from 0-100%. The horizontal blue line corresponds to 65% power and the horizontal red line corresponds to 80% power.
